# One Step Is Not Enough: A Multi-Step Procedure for Building the Training Set of a Query by String Keyword Spotting System to Assist the Transcription of Historical Document

**DOI:** 10.3390/jimaging6100109

**Published:** 2020-10-13

**Authors:** Antonio Parziale, Giuliana Capriolo, Angelo Marcelli

**Affiliations:** 1 Department of Information and Electrical Engineering and Applied Mathematics, University of Salerno, Via Giovanni Paolo II, 132, 84084 Fisciano (SA), Italy; amarcelli@unisa.it; 2 Department of Cultural Heritage, University of Salerno, Via Giovanni Paolo II, 132, 84084 Fisciano (SA), Italy; gcapriolo@unisa.it

**Keywords:** keyword spotting, assisted transcription, handwritten documents, training set, automatic document processing, historical documents, digital transformation, cultural heritage

## Abstract

Digital libraries offer access to a large number of handwritten historical documents. These documents are available as raw images and therefore their content is not searchable. A fully manual transcription is time-consuming and expensive while a fully automatic transcription is cheaper but not comparable in terms of accuracy. The performance of automatic transcription systems is strictly related to the composition of the training set. We propose a multi-step procedure that exploits a Keyword Spotting system and human validation for building up a training set in a time shorter than the one required by a fully manual procedure. The multi-step procedure was tested on a data set made up of 50 pages extracted from the Bentham collection. The palaeographer that transcribed the data set with the multi-step procedure instead of the fully manual procedure had a time gain of 52.54%. Moreover, a small size training set that allowed the keyword spotting system to show a precision value greater than the recall value was built with the multi-step procedure in a time equal to 35.25% of the time required for annotating the whole data set.

## 1. Introduction

In the last decade, significant investments were made for the digital transformation of cultural heritage material. Online digital libraries store and share a huge number of historical books and manuscripts that were scanned for ensuring their preservation along the centuries. These digital collections are not searchable because their documents are digital images. Therefore, these images need to be transcribed in order to allow the indexing and querying of the digital libraries.

A fully manual transcription cannot be a solution because it is a time-consuming and expensive process. In fact, a large number of manuscripts need to be digitized and the trouble in reading documents written with a lexicon different respect to the one used nowadays impose the involvement of highly qualified experts in the transcription process.

On the other hand, a fully automatic transcription is cheaper but not comparable in terms of transcription accuracy. The state-of-the-art technologies for automatic transcription [1,2,3,4] can be grouped into two families: recognition based and recognition free approaches.

Handwritten text recognition (HTR) systems transcribe word images by classifying them, i.e., by recognizing their labels among the terms of a lexicon. These systems are based on Hidden Markov Models [5], neural networks [6] or a combination of different classifiers [7,8] and often rely on a language model [9]. These methods require a large number of annotated word images, which are usually referred to as *training set* (*TS*), for being able to automatically transcribe new word images. The main lack of recognition based systems is that they cannot correctly classify samples in which the transcription is not one of the labels associated to the word images included in the training set. In a common scenario where it is not possible to limit the lexicon of the data collection, HTR technologies show low accuracy values and a heavy human-expert correction work is needed [10].

Keyword spotting (KWS) systems adopt recognition-free methods for retrieving all instances of user queries in a collection of documents [11,12]. When a subject formulates a query the system outputs a ranked list of samples that are more similar to the query. KWS systems are usually grouped into two classes, depending on how the query is represented: query-by-example (QbE) and query-by-string (QbS) systems [1]. QbE systems require that users provide some examples of the word they want to search in the document collection [13,14,15], whereas QbS systems allow to provide a text string, named *keyword*, as query [16,17,18,19]. In the last few years, word spotting systems that can be used with both QbE and QbS search options have been proposed by exploiting, for example, an end-to-end deep neural network architecture [20] or pyramidal histogram of characters embeddings [21,22].

The limit of QbE systems is that users can only search for words that appear at least once in the document collection since an actual instance of the query word is required. On the other hand, QbS systems are the most suitable for transcription of the document because they allow arbitrary textual queries and each keyword can be potentially spotted and eventually transcribed but they have to learn the mapping between textual and visual representations, which is usually achieved through manually annotated training samples [23].

The performance of a KWS system is typically given in terms of recall and precision rates. The recall is the fraction of the total amount of relevant words, i.e., the ones corresponding to the queries, that were retrieved by the system. The precision is the fraction of relevant words among the ones retrieved by the system after a query.

The use of convolutional neural networks [23,24] increased the performance of word spotting systems but these networks need a training set with a large amount of annotated data for being trained. Many solutions have been proposed for improving the word spotting performance without increasing the size of the training set: sample selection [25], data augmentation [23], transfer learning [26,27], training on synthetic data [22,28] and relaxed feature matching [29].

Starting from the idea that combining human validation efforts and automatic systems allows to transcribe documents faster and with the desired level of accuracy [30], KWS systems have been adopted as tools for assisting human beings in the transcription of historical documents [19,31,32]. In such an interactive system the terms already included in the keyword list are used as queries to the KWS. Afterward, the KWS spots the word images that may correspond to the searched terms and, eventually, the user validates the KWS outputs with the result that the images are annotated.

Recently, a performance model for transcription tools equipped with a KWS system and a graphic user interface (GUI) for validating and correcting the KWS system’s outputs has been proposed in [33]. This performance model shows that the advantage of using a KWS for word image transcription strongly depends on the size of the training set and, even more, on the variety of samples that are included. As with regards to the training set arrangement, the performance model indicates that it is necessary to guarantee, for each keyword, a precision value greater than the recall value. In this way, the KWS is capable of spotting at least a few instances of a keyword and there is a benefit in using the system because the time for validating the KWS outputs is less than the time for manually transcribing the documents. Thus, the authors conclude recommending palaeographers to select with great care the word images to include in the training set in order to achieve the required level of precision and recall to make the use of the KWS system profitable for transcribing the documents.

Based on these considerations, we propose a procedure that exploits a KWS as a tool for assisting the palaeographers in building up a training set that fulfills the recommendations of the performance model in [33]. This procedure involves, at the bootstrap, the manual transcription of a small subset of pages to build an initial training set and, consequently, the definition of an initial keyword list. Then, the training set and the keyword list are updated at each step until the precision of the KWS system overcomes the recall. For this purpose, the documents without transcription are divided in batches that are processed by the KWS system. The palaeographers transcribe documents in a batch by searching for all the terms in the keyword list and validating the outputs of the KWS system.

The adoption of this procedure allows to reduce the time required for building up the training set because the samples that are correctly retrieved by the system are transcribed in a time that is shorter than the one required by the manual transcription. Even more, this procedure allows to track the values of precision and recall of the system providing a mechanism for evaluating the goodness of the training set.

The experimental results presented in this paper confirm that the iterative construction of the training set is executed in a time that is significantly lower than the time required for manually transcribing the same data collection.

The remaining of the paper is organized as follows: Section 2 describes the KWS system, the multi-step procedure and the data set adopted for the experimentation, Section 3 compares the human efforts required for building a training set with a manual and an interactive multi-step procedure, Section 4 concludes the paper discussing the results and highlighting the future research steps.

## 2. Materials and Methods

**Figure 1:**
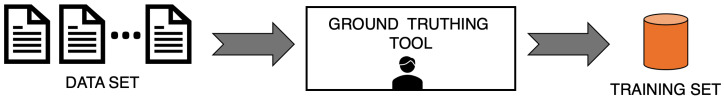
Architecture adopted by many tools for building a training set. Ground truthing tools offer different functionalities for manually segmenting and annotating words contained in the documents belonging to the data set. Usually, human beings annotate all the words contained in the documents without any selection criteria.

Different tools are available for carrying out manuscript transcription, as for example Aletheia [34], a ground truthing tool, and Transkribus [35], a platform for the digitization, transcription, recognition and searching of historical documents. Usually, most of the tools adopt an architecture as the one shown in Figure 1: a collection of documents, the data set *DS*, is manually transcribed and the annotated word images are included in the training set. Platforms as Transkribus use HTR or KWS systems previously trained for annotating new documents and allow to validate the transcriptions at the end of the automatic process.

**Figure 2:**
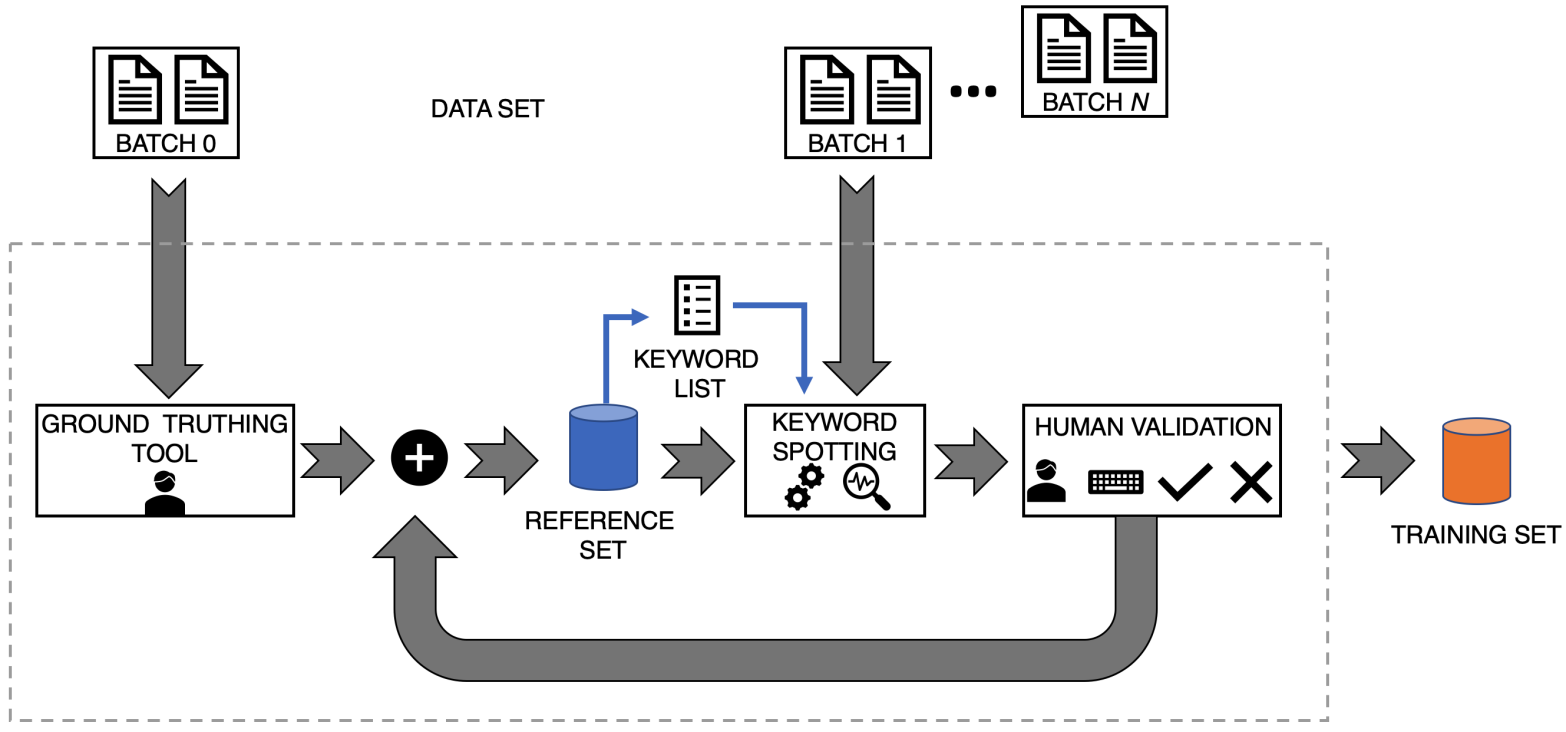
Architecture of a system for building up a training set through a multi-step procedure that interleaves a query-by-string retrieval system and human validation. The data set is divided in batches. Batch 0 is manually transcribed, while the other ones are processed by the keyword spotting (KWS) system and the human validation stage. The keyword list contains the transcriptions of word images included in the reference set with no repetitions. The keyword list is used for querying the KWS system and spotting word images in the batch under analysis. At each step the reference set is updated with the word images of the batch in which the transcription is not yet contained in the keyword list. Thus, at the end of the multi-step procedure, the data set is transcribed and the training set is created.

In this paper document transcription is carried out with a system that stands out from all the others for being based on a multi-step procedure that interleaves a query-by-string retrieval system and human validation [19], as shown in Figure 2. In particular, the system has been designed for pursuing two goals: one is reducing the human time effort for building a *TS* to be used by any HTR or KWS system, the other is to build up a small size training set, from here on called *reference set* (*RS*), used by the KWS system we adopted for the assisted transcription of the *DS*. The *RS* is built by taking care of including samples that are instances of a variety of keywords and aiming for a precision value greater than recall for each keyword. Differently from the *RS*, a *TS* has a bigger size and its samples are collected without any selection criteria.

In the next subsections we briefly summarize the architecture of the proposed system and how it has been used in the experimentation.

### 2.1. Query-by-String Retrieval System

The QbS system used during the experimentation is a segmentation-based keyword spotting system [19] that adopts the algorithm in [36] for extracting word images by any processed document.

Each word is binarized adopting the Otsu method [37] and represented by its skeleton. The trajectory executed by the subject for writing the word is recovered by transforming the word’s skeleton in a graph that is traversed following criteria derived by handwriting generation [38]. Eventually, each trajectory is segmented in elementary movements named strokes [39].

**Figure 3:**
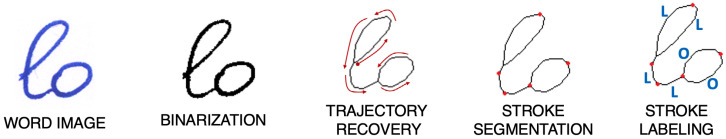
Each word image extracted from a document is processed by the query-by-string (QbS) retrieval system through the following steps: binarization [37], trajectory recovery [38], stroke segmentation [39] and, if a transcription is available for the word image, stroke labeling [40].

When a transcription is available for a word image, as in the case of samples in *RS*, each stroke is labeled with the ASCII code of the character it belongs to [40]. Figure 3 shows how a word image is elaborated by the system.

When a textual query is executed, documents to be transcribed are scanned looking for word images that are instances of the keyword. The trajectory of a word image extracted from one of these documents is compared with all the trajectories stored in *RS* looking for sequences of strokes with similar shapes [41]. When two similar sequences of strokes are found, the transcription associated to the matching strokes belonging to the trajectory in *RS* is assigned to the matching strokes belonging to the trajectory in the document to be transcribed. Because of handwriting variability, different transcriptions could be assigned to the same sequence of strokes.

The ranked list of all the possible interpretations for a word image is obtained by traversing a graph in which the nodes represent the transcriptions associated with the strokes that matched during the comparison with the trajectories in *RS* [42].

When a subject queries for a keyword, the QbS system outputs all the word images in *DS* in which the ranked list of interpretations include the desired keyword.

### 2.2. Multi-Step Procedure for Reference Set Construction

The *RS* is incrementally built by interleaving keyword spotting and human validation. The QbS keyword spotting system described in the previous section is followed by a human validation stage, as shown in Figure 2, with the aim of implementing an interactive multi-step procedure that speeds up the transcription of the *DS*.

The first step of the procedure involves the split of the *DS* in batches and the manual transcription of one of them in order to build the bootstrap *RS*. The unique transcriptions of the word images in *RS* are copied in the keyword list that will be used for submitting a query to the system and spotting words in a new batch.

After the bootstrap, word spotting and human validation are alternated for incrementally updating the *RS* until the precision rate of the system overcomes the recall rate. The *RS* is updated until it is no longer possible to increase the precision with respect to the recall. Afterward, documents that are not yet transcribed are processed in a final step. If the precision never overcomes the recall, the *RS* is updated until the last document is transcribed. The *DS* is fully transcribed and the *TS* is created whether the condition on precision and recall is verified or not. In fact, at the end of each step, all the words included in a batch are transcribed and included in the *TS*.

For each entry in the keyword list, the QbS retrieves all the word images that contain the desired keyword in their ranked list of interpretations. Depending on the performance of the system, the retrieved images can be instances of the desired keyword, instances of other keywords or even instances of terms not included in the keyword list. Word images that are instances of terms not included in the keyword list of the actual step are named *Out-Of-Vocabulary* (*OOV*) words. Eventually, because the KWS has a Recall lower than 1, it could happen that word images that are instances of entries of the keyword list are never retrieved by the system, even after many steps of keyword spotting and validation. These word images are named *missed words*.

**Figure 4:**
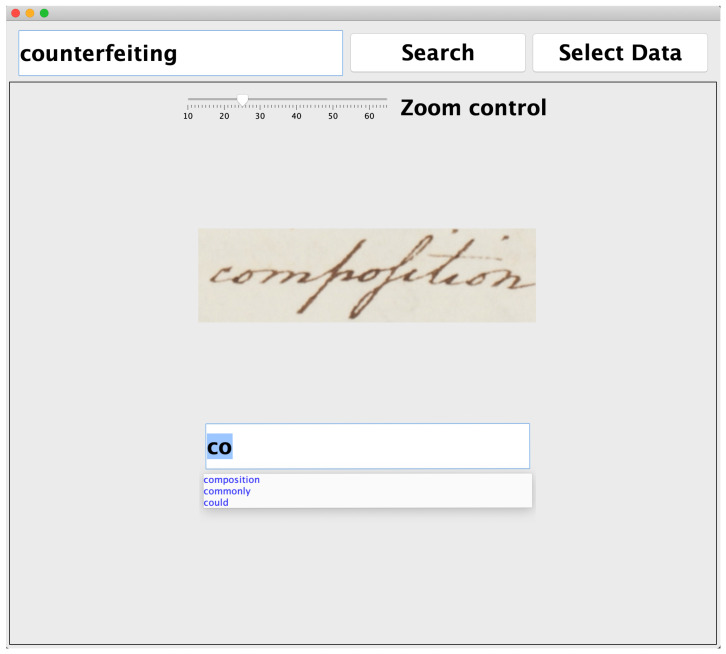
Graphic user interface (GUI) designed for validating the output of the KWS system. The keyword spotted by the system is shown in the text box placed in the top left corner. The button “Search” starts the word spotting. The button “Select Data” allows to select the data set *DS*. The slider regulates the dimension of the image. The text box located on the bottom is used for typing the transcription of the word image. The text box supports the auto-completion mode.

The GUI shown in Figure 4 allows human beings to validate the output of the KWS system by providing two functionalities:

- To confirm with the right click of the mouse the retrieved images that are instances of the query;
- To label a word image by typing its transcription in a text box. For speeding up the typing, the text box works in auto-complete mode by suggesting possible transcriptions taken from the keyword list.

Human validation has the effect of updating the *RS* and keyword list with the annotated *OOV*. The *RS* and keyword list are updated only at the end of a step of the procedure, i.e., when all the entries of the keyword list have been searched in the batch and validated by the human being. The updated *RS* and keyword list are used for spotting new word images in a new batch of documents.

### 2.3. Data Set

The experimentation was carried out on handwritten documents extracted from the Bentham collection, a corpus of documents written by the English philosopher Jeremy Bentham (1748–1832) and his secretaries over a period of sixty years [43]. These handwritten documents have been used in competitions on keyword spotting systems [2,4] and handwritten recognition systems [3].

In particular, the data collection used in the experimentation includes 50 pages that are split into 10 batches of 5 pages. One batch is manually transcribed in order to create the *RS* and the keyword list that will be used during the first step of the KWS system. Batches are comparable in terms of word images and unique words. Table 1 shows the pages assigned to each batch and the number of word images per batch. The bootstrap keyword list contains 354 entries corresponding to the unique words of the bootstrap batch.

### 2.4. Characterization of Human Effort in Transcription

One palaeographer was involved in the experimentation. We asked her to manually transcribe the 5 documents included in the bootstrap batch and to exploit the multi-step procedure and the GUI described in the previous section for transcribing the 45 documents included in the other batches.

The time spent by the palaeographer for manually transcribing the 1089 images included in the bootstrap batch was equal to 10,127.7 s and a single word was transcribed in a mean time ${\bar{T}}_{word}$ equal to 9.3 s.

During the multi-step procedure we recorded the activities executed by the palaeographer for validating and correcting the output of the KWS system. The mean time ${\bar{T}}_{val}$ required for validating a correct retrieved image with a simple mouse click was equal to 1 s. When the system retrieved an image that was an instance of another entry of the keyword list, the palaeographer had to correct the transcription by typing the correct label. Thanks to the auto-complete mode, the palaeographer had to write only the first characters of the actual transcription and the system automatically completed it. Therefore, the mean time ${\bar{T}}_{err}$ required for correcting the transcription of a word that is an instance of a keyword was equal to 5 s. When the system retrieved an *OOV* word, the auto-complete mode did not speed up the manual transcription and the mean time ${\bar{T}}_{OOV}$ was the same as ${\bar{T}}_{word}$.

Eventually, the GUI shows all the word images in which the transcription is empty as they were not retrieved by the system. The missed words, which are images that are instances of the keywords but are without a transcription because the recall is lower than 1, were annotated in a mean time ${\bar{T}}_{miss}$ equal to ${\bar{T}}_{err}$ thanks to the auto-complete mode.

Table 2 reports the means and standard deviations of the times for annotating the word images during the experimentation.

## 3. Results

The experimentation has the aim of evaluating how good the multi-step procedure in building up a training set to be used in a KWS system is for document transcription.

As described in the previous section, the multi-step procedure involves, at each step, the word spotting of all the entries in the keyword list and the validation or transcription performed by a human being. $N_{val}\left(\mathrm{step}\right)$, $N_{err}\left(\mathrm{step}\right)$, $N_{miss}\left(\mathrm{step}\right)$ and $N_{OOV}\left(\mathrm{step}\right)$ are the correct, wrong, missed and *OOV* words processed by the system at the end of each step, respectively. $N_{batch}\left(\mathrm{step}\right)$ is the number of word images processed at each step. Eventually, $KW_{OOV}\left(\mathrm{step}\right)$ is the number of unique transcriptions of the *OOV* word images at each step.

At the bootstrap step (step 0), it is required that the human being manually transcribes all the words in the batch. The word images annotated at step 0 are used for creating the *RS*, which will be updated during the following steps, and their transcriptions, taken once if many words have the same transcription, populate the keyword list.

At each step, each item of the keyword list is used as a query for the KWS system. *OOV* words and their labels are used for updating the *RS* and the keyword list that will be used at the next step. $N_{RS}\left(\mathrm{step}\right)$ and $N_{KL}\left(\mathrm{step}\right)$ are the size of the reference set *RS* and the number of terms in the keyword list at the beginning of each step and they are defined as in Equations (1) and (2), respectively. It is worth noting that at the bootstrap step the keyword list is empty and all the manually transcribed words are considered *OOV* words.

(1) $$N_{RS}\left(\mathrm{step}\right)=\left\{\begin{array}{cc} N_{batch}\left(0\right), & \mathrm{if}\;\mathrm{step}\;\mathrm{is}\;1\\ N_{RS}\left(\mathrm{step}-1\right)+N_{OOV}\left(\mathrm{step}-1\right), & \mathrm{if}\;\mathrm{step}\;>\;1 \end{array}\right.$$

(2) $$N_{KL}\left(\mathrm{step}\right)=\left\{\begin{array}{cc} KW_{OOV}\left(0\right), & \mathrm{if}\;\mathrm{step}\;\mathrm{is}\;1\\ N_{KL}\left(\mathrm{step}-1\right)+KW_{OOV}\left(\mathrm{step}-1\right), & \mathrm{if}\;\mathrm{step}\;>\;1 \end{array}\right.$$

The metrics adopted for evaluating the procedure are defined in Section 3.1 and Section 3.2 reports the comparison between the multi-step procedure and a fully manual transcription.

### 3.1. Metrics

The multi-step procedure is evaluated in terms of time saved to manually transcribe the data set and automatic transcription rate. The procedure is compared with respect to a baseline system that allows the manual transcription of the data set. Although the baseline system does not involve a multi-step procedure, Equation (3) computes the time spent for a fully manual transcription of *DS* as it was executed in more than one step. This formulation allows the comparison between the baseline system and the KWS system.

(3) $$T_{man}\left(\mathrm{step}\right)=\left\{\begin{array}{cc} {\bar{T}}_{word}*N_{batch}\left(0\right), & \mathrm{if}\;\mathrm{step}\;\mathrm{is}\;0\\ {\bar{T}}_{word}*N_{batch}\left(\mathrm{step}\right)+T_{man}\left(\mathrm{step}-1\right), & \mathrm{if}\;\mathrm{step}\;>\;0 \end{array}\right.$$

Equation (4) defines the time spent by a human being for validating with a mouse click ($T_{clk}$) the correct word images retrieved by the system while Equation (5) defines the time spent for labeling ($T_{lab}$) wrong, missed and *OOV* words at each step of the multi-step procedure.

(4) $$T_{clk}\left(\mathrm{step}\right)={\bar{T}}_{val}*N_{val}\left(\mathrm{step}\right)$$

(5) $$T_{lab}\left(\mathrm{step}\right)={\bar{T}}_{err}*N_{err}\left(\mathrm{step}\right)+{\bar{T}}_{OOV}*N_{OOV}\left(\mathrm{step}\right)+{\bar{T}}_{miss}*N_{miss}\left(\mathrm{step}\right)$$

The human time effort for building up the *RS* is computed as in Equation (6). At the bootstrap step, the manual transcription is required for setting up the starting training set and keyword list.

(6) $$T_{hte}\left(\mathrm{step}\right)=\left\{\begin{array}{cc} T_{man}\left(0\right), & \mathrm{if}\;\mathrm{step}\;\mathrm{is}\;0\\ T_{clk}\left(\mathrm{step}\right)+T_{lab}\left(\mathrm{step}\right)+T_{hte}\left(\mathrm{step}-1\right), & \mathrm{if}\;\mathrm{step}\;>\;0 \end{array}\right.$$

As suggested in [44], we introduce Equation (7) for measuring the time gained with the multi-step procedure with respect to the baseline system. Gain(step) could vary between 0% and 100% and it is strongly related to the values of Precision and Recall, defined in Equations (8) and (9), respectively.

(7) $$Gain\left(\mathrm{step}\right)=\left(1-\frac{T_{hte}\left(\mathrm{step}\right)}{T_{man}\left(\mathrm{step}\right)}\right)*100$$

(8) $$\mathrm{Precision}\left(\mathrm{step}\right)=\left(\frac{N_{val}\left(\mathrm{step}\right)}{N_{batch}\left(\mathrm{step}\right)-N_{miss}\left(\mathrm{step}\right)}\right)*100,\;\mathrm{for}\;\mathrm{step}>0$$

(9) $$\mathrm{Recall}\left(\mathrm{step}\right)=\left(\frac{N_{val}\left(\mathrm{step}\right)}{N_{batch}\left(\mathrm{step}\right)-N_{OOV}\left(\mathrm{step}\right)}\right)*100,\;\mathrm{for}\;\mathrm{step}>0$$

Eventually, we introduce other two metrics for evaluating the system: the reference set updating rate ($R_{new}(\mathrm{step})$) and the automatic transcription rate ($R_{auto}\left(\mathrm{step}\right)$), defined by Equations (10) and (11), respectively. $R_{new}(\mathrm{step})$ measures the percentage of manual transcriptions that contribute to the update of the reference set. It corresponds to the percentage of *OOV* words with respect to all the images that are manually transcribed up to the actual step. $R_{auto}\left(\mathrm{step}\right)$ measures the percentage of word images that are correctly transcribed by the KWS system with respect to the images that could be automatically transcribed up to the actual step. If a KWS that never fails was available, both the metrics would be equal to 100: missed and wrong word images would be absent and the human being would manually transcribe only the *OOV* words, which are the words used for updating the *RS*.

(10) $$R_{new}\left(\mathrm{step}\right)=\left(\frac{\sum_{s=1}^{step}N_{OOV}\left(s\right)}{\sum_{s=1}^{step}\left(N_{OOV}\left(s\right)+N_{miss}\left(s\right)+N_{err}\left(s\right)\right)}\right)*100,\;\mathrm{for}\;\mathrm{step}>0$$

(11) $$R_{auto}\left(\mathrm{step}\right)=\left(\frac{\sum_{s=1}^{step}N_{val}\left(s\right)}{\sum_{s=1}^{step}\left(N_{val}\left(s\right)+N_{miss}\left(s\right)+N_{err}\left(s\right)\right)}\right)*100,\;\mathrm{for}\;\mathrm{step}>0$$

### 3.2. Multi-Step Procedure vs. Manual Procedure

Fifty handwritten pages were selected as *DS* to be transcribed for building up a training set. These pages are split into batches, as reported in Table 1.

The multi-step procedure adopts a KWS system for building up an *RS* step by step. Table 3 shows the number of words that are retrieved by the system, the number of words that are missed and how the size of the training set and of the keyword list vary at each step. Table 4 reports the performance of the system in terms of precision, recall, $R_{new}\left(step\right)$ and $R_{auto}\left(step\right)$, at each step. Eventually, Table 5 reports the performance of the system in terms of transcription time.

It is worth noting that the performance of the system at the *i*-th step is obtained with the *RS* rebuilt at the end of the *(i-1)*-th step. For example, the performance at step 2 is obtained on a batch of 1204 word images with an *RS* of 1332 word images and a keyword list of 525 entries. The *RS* used at step 2 is made up of the 1089 word images manually transcribed at step 0 and the 243 *OOV* words transcribed at step 1. The time spent by a human being that uses the GUI described before for building up the *RS* used at step 2 is equal to 14,916.6 s.

The multi-step procedure allows to compute the values of precision and recall that the KWS obtains on a batch of documents with the *RS* built step by step. The best *RS* configuration is the one that allows the KWS to obtain a precision value greater than the recall. Our system reaches that condition at step 7 with a recall equal to 59.62% and a precision equal to 63.33%, as shown in Table 4. At the same step, the KWS system reaches the highest value of automatic transcription rate (57.18%) and the human time effort up to step 6 is equal to 35,223.3 s. These values are obtained on a batch of 1052 word images with a *RS* made up of 2281 word images and a keyword list of 1279 entries. From step 1 to step 6, 33.08% of the words manually annotated by the palaeographer are used for updating the *RS*.

Once the precision overcomes the recall the multi-step procedure ends with a last step on a bigger batch that, in our case, is made up by batch 8 and batch 9 in Table 1. The results show that it is not advantageous to rebuild the *RS* at the end of step 7 because there is a significant reduction of the precision value due to an increase of $N_{err}$.

As with regards to the baseline system, the time required to manually transcribe all the words in 50 pages is 99,919.2 s and the time spent up to batch 6 is 68,922.3 s, as shown in Table 5.

Therefore, by using the multi-step procedure, the palaeographer gained 48.89% of the time with respect to the fully manual procedure for building the *RS* up to step 6 and she gained 52.54% of the time for annotating the whole *DS*.

The last two columns in Table 5 allow to compare the manual and the multi-step procedure in terms of the fraction of time spent in transcribing all the batches with respect to the time required by the fully manual procedure. The multi-step procedure reaches the desired condition of precision greater than recall in a time that is only 35.25% of the total time spent with the manual procedure.

Eventually, we notice that the system shows an automatic transcription rate slightly greater than 50% starting from step 3, when the KWS is equipped with the *RS* built at the end of step 2.

### 3.3. Statistical Analysis

The metrics reported in Table 5, as for example the time spent in document transcription with the manual and the multi-step procedure, depend on the mean time ${\bar{T}}_{word}$ measured when the palaeographer manually transcribed the five pages included in the bootstrap batch.

The value of ${\bar{T}}_{word}$ is computed at the end of the bootstrap step and it can be used for computing $T_{man}\left(\mathrm{step}\right)$ and $T_{hte}\left(\mathrm{step}\right)$ at the following steps if the word length distribution of batches transcribed with the multi-step procedure is equal to the word length distribution of the bootstrap batch. In fact, a variation in the word length distribution would have an effect on the mean word transcription time because the transcription time depends on word length, i.e., the number of characters in the word: the longer the word is, the longer the time required for reading and typing it.

**Figure 5:**
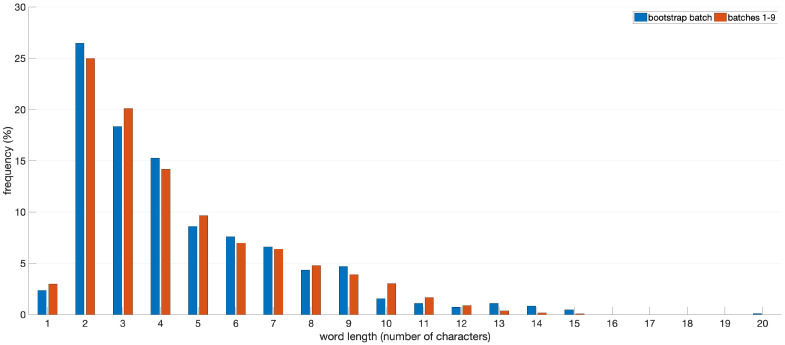
Word length distribution. The distribution of words in the bootstrap batch is depicted in blue. The distribution of words included in the union of batches from 1 to 9 is depicted in red.

Figure 5 shows the word length distribution computed over the bootstrap batch and the union of the other nine batches.

A statistical test was performed in order to verify that word length is equally distributed between the two samples of words and to validate the comparison between the manual and the multi-step procedure.

We tested the null hypothesis $H_{0}$ that both samples have been drawn from the same population with the Epps and Singleton test [45] implemented in SciPy v. 1.5.2. This test does not assume that samples are drawn from continuous distribution and therefore it is suitable for our case. The Epps and Singleton test returned a statistic value equal to 0.1131 and a *p*-value, which gives the probability of falsely rejecting $H_{0}$, equal to 0.998.

This result confirms that word length is equally distributed between the bootstrap batch and the union of the other nine batches and therefore the values of $T_{man}\left(\mathrm{step}\right)$ and $T_{hte}\left(\mathrm{step}\right)$ are valid and can be compared.

### 3.4. Comparison with the State of the Art

HTR and KWS systems have been adopted in different frameworks for transcribing handwritten documents. Human beings and automatic systems work jointly to speed-up the manual transcription process and drastically reduce the cost of training data creation. The performance of the interactive systems for the assisted transcription is usually measured in terms of number of actions executed by the transcriber, as for example keystrokes, in order to correct the automatic transcription. We measured the performance of the system in terms of transcription time because it is a direct measure of the human effort and its cost.

Table 6 lists the papers that adopt the transcription time as a performance measure. It is worth noting that Table 6 does not provide a rank of the systems for the computer-assisted transcription of historical documents. We cannot fairly compare the systems in Table 6 because the expertise of the transcribers and the legibility of collection adopted in each experimentation influence the transcription time. Moreover, papers reported mean transcription times by using different units (seconds per word, seconds per line, seconds per group of lines, etc.) and the times were converted by us in seconds per word adopting the approximation reported in the footnotes of Table 6. Therefore, Table 6 provides a rough indication of the transcription time spent by the palaeographers that adopted one of the listed systems.

The experimental studies presented in [46,47] were performed with the same HTR, which is based on Hidden Markov modes and N-grams models, on two different document collections. The HTR system proposed a full transcript of a given text line image and every time the user amended a wrong word the following ones were updated by the system. In [46] the user was an expert palaeographer while in [47] students in History were involved. In both the papers, the authors noticed a significant typing effort reduction that did not result in a net user time effort savings. This counterintuitive finding was explained by taking into account the additional amount of time the user needed to read and understand each system prediction, which might change after each interaction step.

The study in [48] was conducted by using a QbE system coupled with a relevance feedback mechanism that introduced the human being in the retrieval loop. The transcription time was measured on 50 pages extracted from the Bentham collection. The authors measured a transcription time equal to 9.55 s per word when the documents were automatically segmented in words but manually transcribed, while the time was equal to 4.21 s per word when the interactive system was adopted. Overall, their system allowed to gain 55.9% of the time with respect to the fully manual transcription.

The transcription times measured in [48] are in line with the ones reported in this paper. The difference between the manual transcription times (9.3 s instead of 9.55 s per word) is negligible taking into account that the users involved in the two studies are different. The transcription time we measured with the interactive system is slightly greater than the one reported in [48] (4.41 s instead of 4.21 s per word) if we take into account the time spent for the manual transcription of the bootstrap batch, while is lower (3.86 s instead of 4.21 s per word) if we consider only the steps that exploit our KWS system. In this regard, it is worth noting that the tool based on the QbE system does not require a training step while the transcription times reported in the other two papers [46,47] do not take into account the time spent for training the HTR.

If we compare the four tools without taking into account the time spent for building up the training set, we notice that their transcription times are similar. If we compare the systems in terms of saved time with respect to the manual transcription, our system is the one that allows obtaining the biggest time gain.

## 4. Discussion and Conclusions

QbS systems are adopted as tools for assisting human beings in the transcription of historical documents. These systems are beneficial for transcribing documents when they are equipped with a training set in which the samples are instances of as many keywords as possible in order to reduce the occurrence of *OOV* words [44]. Moreover, these systems are efficient in the automatic transcription of a data collection when their precision value is greater than their recall value.

In this paper, we have compared two procedures for transcribing a collection of handwritten historical documents: the baseline and the multi-step procedure.

The baseline procedure involves the manual transcription of the whole *DS* and therefore it is a time-consuming and expensive process. Moreover, this procedure does not allow to apply any selection criteria during the construction of the training set: all the words in *DS* are labeled and included in *TS*.

The multi-step procedure is based on a loop between a KWS system and a human validation stage. The documents available for the construction of the training set are split into batches that are processed one after the other. This procedure takes advantage of a GUI that reduces the transcription time for the words correctly retrieved by the KWS system or that are wrongly retrieved but are instances of another entry of the keyword list. This procedure allows to build up two sets of annotated word images: the training and the reference set. The training set is exactly the same set of annotated word images that it is built with the manual procedure, but it is obtained faster. The reference set is a smaller set of annotated word images that is built for training the QbS system adopted in our transcription tool. The images included in *RS* are selected with the aim of training the QbS system in a way that it could show a precision rate greater than the recall rate. If that condition is verified, the performance model in [33] guarantees that it is advantageous to use the QbS for the transcription of new documents.

We tested the multi-step procedure on 50 pages extracted from the Bentham collection and during the transcription the condition with a precision value greater than recall value was reached. The system showed an increase in the rate of automatic transcription at each step of the procedure up to the value of 57.18%. The key idea of retraining the QbS system only with the *OOV* retrieved at each step together with the short time required for validating a correctly retrieved word image concur to reduce the human time effort, which is the main cost item in the transcription of manuscripts. The palaeographer had a time gain of 48.89% for building up the *RS* and a time gain of 52.54% for transcribing the whole *DS* by adopting the multi-step procedure instead of the baseline procedure.

If the QbS presented in Section 2 is used both for building a training set and the assisted transcription of a collection of documents, the multi-step procedure allows, at the same time, to select and transcribe the most useful word images to be used for training the system.

What is the best size for a batch is an open question. The procedure is less advantageous if the size of the batches is too big because the number of both the word images in the bootstrap batch and the *OOV* words that need to be manually transcribed increases. On the other hand, if the size of batches is too small the number of missed words could increase and the *RS* and the keyword lists are not significantly updated at each step. The experimentation presented here suggests that the number of *OOV* and missed words per step are markers of how good the multi-step procedure is working. In our experiment, the average sum of *OOV* and missed words per step is around 29% of the batch size.

The future steps will regard the improvement of the KWS system in order to reduce the numbers of wrong words retrieved by the system as well as to reduce the missed words. On the other hand, we will investigate new methods that will allow to discover more *OOV* words at each step. These aspects are of paramount importance for increasing the time gained in the transcription and the rate of automatic transcription.

## Figures and Tables

**Table 1:** Data set organization.

Set	Page IDs	Word Images	Unique Words
Bootstrap Batch	p00, p07, p12, p31, p047	1089	354
Batch 1	p01, p02, p03, p04, p05	944	345
Batch 2	p06, p08, p09, p11, p14	1204	429
Batch 3	p15, p16, p17, p18, p19	830	337
Batch 4	p20, p22, p23, p27, p28	1266	431
Batch 5	p30, p32, p33, p36, p38	1038	398
Batch 6	p39, p41, p42, p43, p44	1040	386
Batch 7	p45, p48, p50, p52, p53	1052	370
Batch 8	p54, p56, p57, p58, p61	1243	444
Batch 9	p62, p63, p66, p67, p68	1038	382

**Table 2:** Means and standard deviations of the times measured during the word transcription.

${\bar{T}}_{\mathrm{word}}\pm \sigma_{\mathrm{word}}$	${\bar{T}}_{\mathrm{val}}\pm \sigma_{\mathrm{val}}$	${\bar{T}}_{\mathrm{err}}\pm \sigma_{\mathrm{err}}$	${\bar{T}}_{\mathrm{OOV}}\pm \sigma_{\mathrm{OOV}}$	${\bar{T}}_{\mathrm{miss}}\pm \sigma_{\mathrm{miss}}$
9.3±2.1 s	1.0±0.3 s	5.0±1.4 s	9.3±2.1 s	5±1.4 s

**Table 3:** Word images processed by the KWS system step by step. Note that at step 8, batch 8 and batch 9 have been merged in one single batch.

Step	$N_{\mathrm{batch}}$	$N_{\mathrm{RS}}$	$N_{\mathrm{KL}}$	$N_{\mathrm{val}}$	$N_{\mathrm{OOV}}$	$N_{\mathrm{err}}$	$N_{\mathrm{miss}}$	${\mathrm{KW}}_{\mathrm{OOV}}$
0	1089	-	-	-	-	-	-	354
1	944	1089	354	244	243	314	143	171
2	1204	1332	525	484	239	337	144	190
3	830	1571	715	444	180	116	90	140
4	1266	1751	855	575	191	380	120	142
5	1038	1942	997	634	171	130	103	139
6	1040	2113	1136	529	168	259	84	143
7	1052	2281	1279	570	96	234	152	86
8	2281	2377	1365	899	231	972	179	184

**Table 4:** Recall, Precision, *RS* updating rate and automatic transcription rate evaluated step by step for the tool equipped with the KWS system.

Step	Recall	Precision	$R_{\mathrm{new}}$	$R_{\mathrm{auto}}$
0	-	-	-	-
1	34.81%	30.46%	34.71%	34.81%
2	50.16%	45.66%	33.94%	43.70%
3	68.31%	60.00%	36.66%	50.60%
4	53.49%	50.17%	34.16%	51.52%
5	73.13%	67.81%	35.30%	55.92%
6	60.67%	55.33%	34.94%	56.73%
7	59.62%	63.33%	33.08%	57.18%
8	43.85%	42.77%	28.79%	53.82%

**Table 5:** Transcription time with the baseline system and the multi-step procedure, time gain, step by step.

Step	$T_{\mathrm{clk}}$	$T_{\mathrm{lab}}$	$T_{\mathrm{man}}$	$T_{\mathrm{hte}}$	Gain	$T_{\mathrm{man}}\left(\mathrm{step}\right)/T_{\mathrm{man}}\left(8\right)$	$T_{\mathrm{hte}}\left(\mathrm{step}\right)/T_{\mathrm{man}}\left(8\right)$
0	-	10,127.7	10,127.7	10,127.7	0.00%	10.14%	10.14%
1	244	4544.9	18,906.9	14,916.6	21.10%	18.92%	14.93%
2	484	4627.7	30,104.1	20,028.3	33.47%	30.13%	20.04%
3	444	2704	37,823.1	23,176.3	38.72%	37.85%	23.20%
4	575	4276.3	49,596.9	28,027.6	43.49%	49.64%	28.05%
5	634	2755.3	59,250.3	31,416.9	46.98%	59.30%	31.44%
6	529	3277.4	68,922.3	35,223.3	48.89%	68.98%	35.25%
7	570	2822.8	78,705.9	38,616.1	50.94%	78.77%	38.65%
8	899	7903.3	99,919.2	47,418.4	52.54%	100.00%	47.46%

**Table 6:** Comparison of systems for the assisted transcription of historical documents. Transcription time is measured in seconds per word. Values reported in this table are computed starting from the data reported in each paper as described in the footnotes.

Ref.	Transcription Time		*Gain*	System	Collection
	Manual	Assisted			
	Transcription	Transcription			
[46] ^1^	3.66	3.61	1.36%	HTR	Spanish marriage license books
[47] ^2^	2.78	3.71	−33.4%	HTR	Historia de las plantas
[48]	9.55	4.21	55.9%	QbE	Bentham
*this paper*	9.3	3.86 ^3^	58.5%	QbS	Bentham
		4.41 ^4^	52.54%		

^1^ A set of 116 license records containing 3609 words was manually transcribed in 1.9 min per license record. A set of 117 records containing 3497 words was transcribed with the interactive system in 1.8 min per license record. Time values do not take into account the time spent for training the HTR. ^2^ The transcription time averaged over the 15 weeks of experimentation is around 37 s per line with the interactive system and 27.66 s with the manual transcription. The data set is made up of 19,764 lines and 196,858 words. Time values do not take into account the time spent for training the HTR. ^3^ Mean transcription time computed without taking into account the time spent for transcribing words during the bootstrap step. 9655 words were transcribed in 37,290.7 s. ^4^ Mean transcription time computed by including the time spent for transcribing words during the bootstrap step. 10,744 words were transcribed in 47,418.4 s.

## Abbreviations

The following abbreviations are used in this manuscript:

KWS	keyword spotting systems
QbS	query-by-string
QbE	query-by-example
*OOV*	out-of-vocabulary
*TS*	training set
*RS*	reference set
*DS*	data set
GUI	graphic user interface
